# Investigating the Best Practices for Engagement in Remote Participatory Design: Mixed Methods Analysis of 4 Remote Studies With Family Caregivers

**DOI:** 10.2196/60353

**Published:** 2024-12-03

**Authors:** Anna Jolliff, Richard J Holden, Rupa Valdez, Ryan J Coller, Himalaya Patel, Matthew Zuraw, Anna Linden, Aaron Ganci, Christian Elliott, Nicole E Werner

**Affiliations:** 1 Department of Health & Wellness Design School of Public Health - Bloomington Indiana University Bloomington Bloomington, IN United States; 2 Department of Systems and Information Engineering University of Virginia Charlottesville, VA United States; 3 Department of Public Health Sciences University of Virginia Charlottesville, VA United States; 4 Department of Pediatrics School of Medicine and Public Health University of Wisconsin - Madison Madison, WI United States; 5 Health Systems Research Center for Health Information and Communication Richard L Roudebush Veterans Affairs Medical Center United States Department of Veterans Affairs Indianapolis, IN United States; 6 CareVirtue Technologies San Diego, CA United States; 7 Department of Industrial & Systems Engineering University of Wisconsin - Madison Madison, WI United States; 8 Department of Visual Communication Design Herron School of Art & Design Indiana University Indianapolis Indianapolis, IN United States

**Keywords:** user-centered design, family caregivers, mobile health, digital health, web-based intervention, stakeholder engagement, patient engagement, community-based participatory action research, community participation, qualitative evaluation

## Abstract

**Background:**

Digital health interventions are a promising method for delivering timely support to underresourced family caregivers. The uptake of digital health interventions among caregivers may be improved by engaging caregivers in participatory design (PD). In recent years, there has been a shift toward conducting PD remotely, which may enable participation by previously hard-to-reach groups. However, little is known regarding how best to facilitate engagement in remote PD among family caregivers.

**Objective:**

This study aims to (1) understand the context, quality, and outcomes of family caregivers’ engagement experiences in remote PD and (2) learn which aspects of the observed PD approach facilitated engagement or need to be improved.

**Methods:**

We analyzed qualitative and quantitative data from evaluation and reflection surveys and interviews completed by research and community partners (family caregivers) across 4 remote PD studies. Studies focused on building digital health interventions for family caregivers. For each study, community partners met with research partners for 4 to 5 design sessions across 6 months. After each session, partners completed an evaluation survey. In 1 of the 4 studies, research and community partners completed a reflection survey and interview. Descriptive statistics were used to summarize quantitative evaluation and reflection survey data, while reflexive thematic analysis was used to understand qualitative data.

**Results:**

In 62.9% (83/132) of evaluations across projects 1-3, participants described the session as “very effective.” In 74% (28/38) of evaluations for project 4, participants described feeling “extremely satisfied” with the session. Qualitative data relating to the engagement context identified that the identities of partners, the technological context of remote PD, and partners’ understanding of the project and their role all influenced engagement. Within the domain of engagement quality, relationship-building and co-learning; satisfaction with prework, design activities, time allotted, and the final prototype; and inclusivity and the distribution of influence contributed to partners’ experience of engagement. Outcomes of engagement included partners feeling an ongoing interest in the project after its conclusion, gratitude for participation, and a sense of meaning and self-esteem.

**Conclusions:**

These results indicate high satisfaction with remote PD processes and few losses specific to remote PD. The results also demonstrate specific ways in which processes can be changed to improve partner engagement and outcomes. Community partners should be involved from study inception in defining the problem to be solved, the approach used, and their roles within the project. Throughout the design process, online tools may be used to check partners’ satisfaction with design processes and perceptions of inclusivity and power-sharing. Emphasis should be placed on increasing the psychosocial benefits of engagement (eg, sense of community and purpose) and increasing opportunities to participate in disseminating findings and in future studies.

## Introduction

### Digital Health Interventions for Family Caregivers

The United States is home to 44 million family caregivers of adults with chronic illness, and 11 million of these family caregivers care for someone with Alzheimer disease and related dementias (ADRD) [1,2]. A total of 1% of children in the United States are medically complex, meaning they have multiple chronic illnesses, have functional limitations, and depend on medical technologies for survival [3]. Caregivers of these children and people living with ADRD are often undersupported and underresourced in their role of providing care at home and in their communities, which can lead to caregiver burnout and increased risk for chronic health disorders [4-7]. Digital health interventions that deliver support and education for caregivers are a promising area of research. Digital interventions, by virtue of their remote nature and powerful functionality, have the potential to reduce costs and time associated with traveling to community resources, quickly synthesize resource availability, provide training to caregivers in rural and underresourced areas, and enable connection between isolated caregivers [8]. However, among caregivers of adults, uptake has been hampered when interventions are inaccessible, unhelpful, or hard to use by their target population [8]. Among parent caregivers, ease of use, customizability, and cultural appropriateness have been key to driving uptake [9]. There is a persistent need to ensure that the design of digital health interventions is tailored to these key target populations. To this end, this study aimed to understand how to best facilitate engagement in remote participatory design (PD) so that high-risk populations can be involved in designing the digital health interventions intended to meet their needs.

### PD to Meet Caregiver Needs

The uptake of digital health interventions among caregivers may be improved by engaging caregivers in the design of these interventions. PD is an established strategy for engaging community and academic partners in intervention design [10]. PD is a design process in which academic researchers and software developers partner with those who perform a certain type of work to design a tool, technology, or workflow to support that work [10]. Family caregiving can be understood as work, insofar as multiple people coordinate to perform complex tasks (eg, medication management and symptom tracking) using specialized tools in a particular context [11]. By performing their work, caregivers accrue implicit and tacit knowledge, or work-related knowledge that is highly practical, mostly invisible, and that can be difficult to articulate [12,13]. Because caregivers may not themselves have the skills required to design health intervention technologies, the partnership between caregivers and experts in software development, human-computer interaction, and systems design is key to designing digital interventions that are useful and effective [10,14,15].

### Evaluating Caregiver Engagement in PD

Previous research has involved family caregivers in PD of digital health interventions [16,17]. However, beyond providing a description of the design activities used and the final prototype, the engagement of family caregivers and subject matter experts collaborating in the PD of health information technology has rarely been analyzed, either qualitatively or quantitatively [18,19]. Thus, while we know family caregivers can be incorporated into design processes, we do not know whether the processes used are perceived as effective, inclusive, positive, and impactful by the caregivers involved.

One approach to studying PD for caregivers is to investigate their engagement in PD using the Patient-Centered Outcomes Research Institute (PCORI) theory of action [20]. This framework describes how domains, such as engagement context, engagement activities, and engagement quality, shape outcomes for not just the research but the partners (eg, caregivers) involved. Engagement context is multifaceted, referring to the characteristics of the research team (referred to as *research partners* within the framework) and community members (referred to as *community partners*). Engagement context may include the demographic characteristics, lived experiences, sociohistorical context, feelings of trust, and sense of preparedness to engage in research that partners bring to the design process. Engagement context also includes properties of the design environment, whether remote or in-person. Engagement activities describe the actions taken by research and community partners during the research process, while engagement quality captures research and community partners’ *perceptions* of the research process, including their satisfaction with it. Research outcomes include short- and long-term effects of engagement, such as the release and uptake of findings, and partner outcomes describe how participating in research affected partners, including costs of and benefits to participation. The theory of action can be used to understand ways in which the engagement of caregivers in PD embodies (or fails to embody) engagement ideals.

### Evaluating PD in Remote Contexts

PD has historically occurred in the context of in-person workshops, which may include activities such as affinity diagramming, paper prototyping, and working in breakout groups [18]. However, in recent years, there has been a shift toward conducting PD in online or remote environments [21-24]. For family caregivers, the benefits of this shift are clear: caregivers frequently care for someone who cannot be left alone, have limited time to engage in research, and may not always live close to universities or other research institutions [23,25]. The benefits were magnified during the COVID-19 pandemic, when contagion was of particular concern for caregivers of high-risk populations, such as people living with dementia and children with medical complexity [26]. Challenges to using a remote over in-person PD approach include the unequal distribution of and familiarity with technology across subgroups, the need to adapt traditionally in-person design activities to the remote context, and the difficulty of trust- and rapport-building in the remote setting [21,27,28]. There is a need to learn how to best facilitate engagement in remote PD so that high-risk populations can be involved in the design of the digital health interventions intended to meet their needs.

### Study Objective

In this study, we analyzed qualitative and quantitative data from evaluation and reflection surveys across research and community partners in 4 separate remote PD studies, all of which focused on building digital health interventions that addressed critical issues for caregivers. Community partners were family caregivers of high-risk populations, including caregivers of persons living with dementia and caregivers of children with medical complexity; members of community organizations who serve caregivers; and medical experts. Research partners were members of the research team who assisted with the facilitation of PD sessions. This analysis aimed to (1) understand the context, quality, and outcomes of partners’ engagement experiences in remote PD and (2) learn which aspects of the observed PD approach facilitated engagement or need to be improved going forward.

## Methods

### Overview of Projects

Evaluation and reflection data came from community and research partners in a convenience sample of 4 PD projects conducted remotely between 2021 and 2023. Each project had a distinct purpose and included different research and community partners from the others. The goal of the CareVirtue Planner (Whiplash Technologies; project 1) was to design a website that could assist caregivers of people living with ADRD in the process of financial and legal planning [22]. The goal of Alzheimer’s Family Connection (project 2) was to design a website that could provide resources and connections for rural caregivers of people living with ADRD. The goal of MedS@HOME (project 3) was to design a mobile app that could promote safe medication management among caregivers of children with medical complexity [29]. Finally, the goal of Helping the Helpers (project 4) was to design a technology that could assist caregivers of people living with dementia with medication management at home [30]. This study is a primary analysis of evaluation and reflection data from these 4 projects that have never been published.

### Ethical Considerations

Each of the 4 projects, including their evaluation and reflection components, was considered minimal risk human subjects research and was subject to an expedited ethics review by the institutional review board at the relevant institution (Indiana University Bloomington or University of Wisconsin-Madison; project 1: 16227; project 2: 16214; project 3: 16293 project 4: 16063). Each of these studies received a waiver of signed consent from the institutional review board and instead used a study information sheet that outlined all known risks and protections against risks. Potential participants were then provided with the opportunity to ask questions and to provide verbal consent before participation. All data were deidentified. In projects 1, 2, and 4, community partners were compensated with a US $50 e-gift card after each session. For project 3, participants received US $25 per session and were mailed a check after the conclusion of all sessions.

### Research Partners

Research partners included principal investigators as well as research coordinators, research specialists, and software engineers working on investigators’ grants. Areas of academic and professional expertise included human factors engineering, pediatric medicine, gerontology, counseling psychology, software development, visual communication design, and business development. Project 1 and 2 design sessions were facilitated by author MZ (caregiver support officer, caregiver lived experience); project 3 by NEW (cognitive psychology; associate professor); and project 4 by AG (visual communication design; associate professor) and HP (health informatics). Community and research partners were largely unknown to each other before recruitment and enrollment. In the first design session for each project, the facilitator shared briefly about their personal and professional interests in the research.

### Recruitment of Community Partners

For projects 1, 2, and 4, community partners (caregivers of people living with ADRD or organizations that serve these caregivers) were recruited via email through our research team’s caregiver registry, community agencies (such as aging and disability resource centers and local chapters of the Alzheimer’s Association), and our strategic advisory boards. For project 3, community partners (primary or secondary caregivers of children with medical complexity and medical experts) were recruited from a local pediatric complex care program via email and postal mail. Recruitment included convenience and snowball sampling methods. For projects 1, 2, and 4, no community partners dropped out after enrolling; for project 3, information about dropouts had been destroyed at the time of analysis to meet confidentiality requirements and is, therefore, unavailable.

### Remote Context

All sessions were recorded (with participants’ consent) and took place via Zoom (Zoom Video Communications) or Webex (Cisco Systems). All partners were able to unmute themselves and use the chat feature freely. Partners were encouraged, but not required, to keep their cameras turned on. All research partners were granted cohost capabilities to assist with the technical aspects of the meeting.

### Study Procedure

For all projects, community partners met with the research partners for 4 to 5-hour-long sessions across 6 months. The agenda for each session was guided by our research team’s predefined, published 5-stage process for cocreating a prototype [31]. Sessions focused on problem identification, solution generation, convergence around a single solution, prototyping, and initial evaluation. An example facilitator question related to problem identification is “Where can things go wrong with [your caregiving work]?” and a corresponding question related to solution generation is “What have you done to keep things from going wrong?” After each session, community partners completed an evaluation survey. For project 1, after completing the 5-stage process, volunteering community and research partners completed a reflection survey and interview.

### Data Collection

#### Overview

All evaluation and reflection data were deidentified and stored on an encrypted, cloud-based drive. All evaluation and reflection questions were optional and could be skipped. Because there was no incentive to complete surveys and only meeting participants could access the survey links, it was determined that no additional measures were needed to prevent duplicate entries.

#### Evaluation Data

Immediately after each design session, community partners completed an evaluation survey on Qualtrics (Qualtrics International Inc) software [32]. Survey links were shared via the Zoom or Webex chat feature, and partners could also request that the link be sent via email. In project 4, research partners also completed evaluation surveys. Evaluation surveys were constructed with the intent to understand partners’ satisfaction with the sessions, their perceptions of session effectiveness, and any changes that should be implemented in future sessions. Partners were asked to provide a quantitative rating of the session and qualitative responses to open-ended questions. An example quantitative question was, “On a scale from 1 to 5, how would you rate the effectiveness of the design session?” An example qualitative question was, “What would you suggest we do differently for the next sessions?” Table 1 provides all the quantitative and qualitative evaluation questions asked after each design session for all projects. For projects 1 to 3, all questions appeared on a single survey page, whereas in project 4, questions were spread across 3 survey pages and a “back” button was enabled.

**Table 1:** Quantitative and open-ended questions asked in evaluation surveys.

Participatory design project	Partners completing evaluations	Quantitative evaluation questions	Open-ended evaluation questions
Project 1: CareVirtue Planner (Whiplash Technologies)	ADRD^a^ caregivers	On a scale from 1 to 5, how would you rate the effectiveness of the design session?^b^	What are your thoughts on the effectiveness? What would you suggest we do differently for the next sessions? Is there anything else you want to add?
Project 2: Alzheimer’s Family Connection	Rural ADRD caregivers Community organizations that serve rural ADRD caregivers	On a scale from 1 to 5, how would you rate the effectiveness of the design session?^b^	What are your thoughts on the effectiveness? What would you suggest we do differently for the next sessions? Is there anything else you want to add?
Project 3: MedS@ Home	CMC^c^ caregivers Secondary CMC caregivers Medical experts	On a scale from 1 to 5, how would you rate the effectiveness of the design session?^b^	What are your thoughts on the effectiveness? What would you suggest we do differently for the next sessions? Is there anything else you want to add?
Project 4: Helping the Helpers	ADRD caregivers Research partners	Overall, how satisfied are you with today’s design session?^d^ Did this session make you feel worse or better about the remaining sessions?^e^	What would you need to improve your satisfaction with the design sessions? What made today’s session more effective? What made today’s session less effective? What could be done to better reach goals or make expectations clearer? Do you have any comments or suggestions about the session that you did not get a chance to share today?

^a^ADRD: Alzheimer disease and related dementias.

^b^The questions were scaled from 1 to 5: not effective, somewhat effective, neutral, mostly effective, and very effective.

^c^CMC: children with medical complexity.

^d^The questions were scaled from 1 to 5: extremely dissatisfied, somewhat dissatisfied, neither satisfied nor dissatisfied, somewhat satisfied, and extremely satisfied.

^e^The question were scaled from 1 to 5: much worse, somewhat worse, about the same, somewhat better, and much better.

#### Reflection Data

For project 1, after all the co-design sessions had been completed, research and community partners were offered the opportunity to complete a reflection interview with a staff member who was not a research partner and a reflection survey via Qualtrics. Interviews were recorded, took place on Zoom, and lasted no longer than 30 minutes. Links to the reflection survey were shared in the chat following the interview. The reflection interview guide and survey were coconstructed by research partners on project 1. An example reflection interview question was, “How did your expectations compare/contrast with your actual experience in the design sessions?” In an example quantitative reflection survey question, partners were asked to rate the following statement on a scale of 1 to 100: “My own participation in the design sessions influenced the design of the product.” Questions were spread across 3 survey pages and going “back” was not possible. Textbox 1 presents all questions asked in the reflection interview and survey.

**Quantitative and open-ended reflection questions asked after project 1.**

**Partners completing reflection**

- Caregivers of people with Alzheimer disease or related dementias
- Research partners

**Quantitative questions**

Drag along the slider scale (1-100) to show the extent to which the following statements are true for you:

- My own participation in the design sessions influenced the design of the product.
- The participation of caregivers influenced the design of the product.
- The participation of the research team members influenced the design of the product.

**Open-ended questions (interview)**

- What expectations did you have for the design process before the first design session?
- How did these expectations compare and contrast with your actual experience in the design sessions?
- What expectations did you have for the legal and financial planning tool before the first design session?
- How did these expectations compare and contrast to the ultimate prototype?
- How do you feel your participation in the design sessions influenced product design?

**Open-ended questions (survey)**

- Please elaborate on how you think caregiver participation did or did not influence the design of the product.
- Please elaborate on how you think research team member participation did or did not influence the design of the product.

### Data Analysis

Descriptive statistics were used to summarize quantitative evaluation and reflection survey data from research and community partners. For the preparation of interview data, 2 research team members (AJ and AL) separately watched each reflection interview and took notes on the content. It was not presumed that research team members’ notes could be identical but rather that the use of 2 observers could yield additional and unique observations from which meaning could be derived [33]. The interview notes that did not appear to constitute feedback were excluded from coding. For the preparation of qualitative evaluation and reflection survey data, partner responses that were not believed to constitute feedback (eg, the partner had written “not applicable” or “no opinion”) were excluded from coding. These excluded data were reviewed by a senior researcher with more training in reflexive thematic analysis (NEW) to decrease the likelihood that meaning relevant to the research questions was being dismissed. Incomplete responses (eg, if a partner answered only 1 of the 3 open-ended questions) were retained for analysis. Each response was a single unit of analysis, and responses could be and often were coded to multiple themes.

Evaluation and reflection survey data were combined with interview notes and exported into Excel (Microsoft Corporation). One research team member (AJ) reviewed all the data line-by-line and grouped data by 3 PCORI domains, including engagement context, engagement quality, and partner outcomes. This grouping was completed with constant reference to PCORI’s verbatim definitions of each domain. Following this grouping, we adopted the “Big Q” approach of reflexive thematic analysis by Braun and Clarke [34]. Thus, an objective or single “correct” interpretation of the data was not presumed to be possible. Rather, it was expected that coding would result in an interpretive story about each PCORI domain. Themes were created, iterated upon, and constantly examined for apparent fit to previously coded data.

The primary coder (AJ) was a licensed mental health counselor with training in interpersonal process groups. Facilitators of these types of groups are trained to be mindful of certain risks to group work, including insufficient preparation and imbalances of power, as well as certain benefits, including self-exploration and a sense of connectedness to other group members [35]. The primary coder was present for many, but not all, of the PD sessions and held a junior status on the research team and a nonfacilitative role in sessions. These aspects of the primary coder’s positioning inevitably shaped their interpretation of data. It is not expected that a different coder in a different context would obtain identical results; however, the thick descriptions of our data collection and analytic processes provided in this manuscript may enable others to critically analyze how we arrived at these results and understand the applicability of these results in other contexts.

The final codebook was presented to senior researchers with training in reflexive thematic analysis (NEW and RV) and research partners on projects 1 to 4 to elicit their feedback. The goal of this process was not to rid the codebook of “bias” or the primary coder’s perspective. Rather, this team-wide review enabled crystallization, an alternative to triangulation that seeks comprehensiveness and depth over convergence or consensus [36,37]. Because many of the authors of this manuscript were also research partners in the projects described, the cowriting of results allowed many opportunities for member reflections, which is an alternative to member checking in which participants offer additional, unique insight rather than confirming or denying that the data have been interpreted “correctly” [33].

## Results

### Quantitative Results

#### Overview

A total of 43 community partners and 19 research partners were involved in 31 PD sessions. Within these samples, a subset of 43 (100%) community partners and 2 (10%) research partners completed 170 evaluation surveys. Partners in project 1 completed 32.9% (56/170) of the total surveys, project 2 completed 21.8% (37/170), project 3 completed 22.9% (39/170), and project 4 completed 22.4% (38/170). A subset of 14 partners (7/43, 16% of community partners and 7/19, 37% of research partners) completed a reflection interview and survey. Table 2 presents the demographic information for community and research partners.

**Table 2:** Demographic information for community and research partners.

Characteristics			Community partners (n=43)		Research partners (n=19)
Age (y), mean (SD)			52.3 (15.9)		36.6 (10.1)
**Gender, n (%)**					
	Women	31 (72)		11 (58)	
	Men	12 (28)		7 (27)	
	Nonbinary	0 (0)		1 (5)	
**Race and ethnicity, n (%)**					
	Asian	1 (2)		1 (5)	
	Black	2 (5)		0 (0)	
	Hispanic	1 (2)		0 (0)	
	Multiracial	1 (2)		0 (0)	
	White	35 (81)		18 (95)	
	Other	3 (7)		0 (0)	

#### Quantitative Evaluation and Reflection Data

In 62.9% (83/132) of evaluations across projects 1-3, participants described the session as “very effective.” In 74% (28/38) of evaluations for project 4, participants described feeling “extremely satisfied” with the session. The mean effectiveness and satisfaction ratings ranged between 4 and 5 for all design sessions. Table 3 shows the mean effectiveness, satisfaction, and feeling across sessions.

Community partners rated their own influence and the influence of other co-designers on the final design at an average of 88.8 (SD 14.7) and rated the research team’s influence at 82.4 (SD 24.5) on a scale of 1 to 100. Research partners rated community partner’s influence at an average of 82.6 (SD 21.7) and their own influence and that of other research partners at 64.3 (SD 37.5) on the same scale of 1 to 100.

**Table 3:** Quantitative evaluations of design sessions with time.

Evaluation question	Session 1 (n=7), mean (SD)	Session 2 (n=7), mean (SD)	Session 3 (n=6), mean (SD)	Session 4 (n=6), mean (SD)	Session 5 (n=5), mean (SD)
On a scale from 1 to 5, how would you rate the effectiveness of the design session?^a^ (projects 1-3)	4.4 (0.8)	4.7 (0.6)	4.9 (0.3)	4.8 (0.4)	4.9 (0.2)
Overall, how satisfied are you with today’s design session?^b^ (project 4)	4.4 (0.8)	4.9 (0.4)	4.6 (0.5)	4.4 (1.4)	4.9 (0.4)
Did this session make you feel worse or better about the remaining sessions?^c^ (project 4)	3.75 (1.0)	4.6 (0.5)	4.1 (1.0)	4.4 (0.9)	—^d^

^a^The questions were scaled from 1 to 5: not effective, somewhat effective, neutral, mostly effective, and very effective.

^b^The questions were scaled from 1 to 5: extremely dissatisfied, somewhat dissatisfied, neither satisfied nor dissatisfied, somewhat satisfied, and extremely satisfied.

^c^The questions were scaled from 1 to 5: much worse, somewhat worse, about the same, somewhat better, and much better.

^d^Not applicable.

### Qualitative Results

#### Overview

After subtracting 56 noncoded responses, partners provided 676 free-text responses to evaluation surveys, reflection surveys, and interview notes. Within these responses, 13.5% (91/676) were categorized as nonspecific positive feedback and were not believed to fit an additional theme. In total, 17 themes were created across the 3 PCORI domains of engagement context, engagement quality, and partner outcomes.

#### Nonspecific Positive Feedback

In response to the free-text evaluation questions, partners often responded with brief, positive answers (nonspecific positive feedback). When asked their thoughts on the effectiveness, some partners provided responses such as “great,” “wonderful,” and “all good so far.” When asked what improvement they would like to see in future sessions, some partners responded with “nothing,” and when asked whether they had any remaining comments or suggestions, they often said “no.”

#### Engagement Context

We constructed 4 themes within engagement context: identity influence, technological context, project understanding, and role understanding. Table 4 presents the themes, definitions, and example quotations relating to engagement context.

Partners perceived some aspect of their identity (eg, the status of being a sexual or gender minority individual, the generational identity of being a “millennial,” or a career as a publisher) as influencing their engagement (identity influence). Partners also described the remote context of PD as shaping their expected or actual experience (eg, feeling wary of using videoconferencing software and limitations of remote PD in contrast with in-person PD; technological context). One research partner noted that the process was “less hands on” than traditional PD and wondered how to achieve this going forward in a remote context.

Two context themes pertained to whether partners felt prepared to engage in research, including project understanding and role understanding. Research and community partners alike described the project as different from or consistent with their expectations, reflecting the presence or absence of project understanding before engaging in co-design. Partners also expressed confusion with aspects of the projects once they began, such as what they were designing and how they would design it, reflecting the absence of project understanding once the project was underway. Partners expressed either confusion or understanding pertaining to their role (eg, 1 research partner noted being “less involved than I expected to be”) and expressed perceptions of their role that were sometimes contradictory and sometimes consistent with those of their peers (role understanding).

**Table 4:** Engagement context^a^ themes, definitions, and examples.

Theme	Definition	Examples
Identity influence	A partner perceives one of their identities or traits as influencing their engagement in the project.	“As a sexual and gender minority, some legal and financial processes as a caregiver are more complicated. I was able to bring that perspective.” [Research partner’s interview note] “Background in publishing, experience brainstorming designs and content” [Community partner’s interview note]
Technological context	A partner perceives the remote context of co-design as a facilitator or barrier to engagement.	“I suffer from ‘technostress’ - I thought it would be boring, people wouldn’t share.” [Community partner’s interview note] “One loss of digital platforms- can't have two cooks in the kitchen (can't have side conversations, less potential for multiple people to get involved).” [Research partner’s interview note]
Project understanding	A partner describes the project as consistent with or different from their expectations or describes having no expectations for the project, and a partner expresses understanding or confusion regarding some aspect of the project.	“I think the goals and expectations are clear!” [Community partner’s evaluation survey] “I expected to pitch ideas, make sketches, draw on the whiteboard. It was less hands on than that.” [Research partner’s interview note] “I think that there’s a disconnect between those working on developing the app and us who see the app. For those developing it, many things might seem intuitive as they will understand what’s coming next, but for us, who see the app and can only use the functions highlighted in blue at this point, it’s less intuitive.” [Community partner’s evaluation survey]
Role understanding	A partner expresses understanding or confusion about their role, and partners express contradictory perceptions regarding their role.	“Thought research team was going to be much more responsible for keeping things going- taking notes, making agendas- basically leading it. and we weren’t.” [Research partner’s interview note] “In a traditional co-design process, I would have expected that I was as much a co-designer as anyone else. It felt like if I was getting involved in this process, I would have felt like I was taking space away from the participants.” [Research partner’s interview note]

^a^Engagement context: “Resources and circumstances surrounding the practice of engagement in research that may affect how engagement occurs and its impact” [20].

#### Engagement Quality

We constructed 8 themes within engagement quality: relationship-building, co-learning, desire for prework, satisfaction with design activities, satisfaction with the time allotted, satisfaction with the final tool, influence, and inclusivity. Table 5 presents the themes, definitions, and example quotations relating to engagement quality.

Community partners commented on their appreciation of other group members (eg, their contributions and demeanor; relationship-building) and reported learning from other group members (eg, about a caregiver resource; co-learning). Partners also described feeling satisfied or dissatisfied with various aspects, including prework (eg, appreciating the work assigned before sessions or wanting more of it), the design activities (eg, commenting that activity was effective or suggesting a different activity), the time allotted to complete activities (eg, saying that there was not enough time), and the prototype (eg, appreciating its functionality or wishing it were more complete).

Partners shared both positive and negative perceptions of decision-making processes, including the extent to which decisions were guided by research partners versus community partners (influence). Furthermore, partners commented on the extent to which they felt empowered to contribute, actually did contribute, or perceived contributions as equal across partners (inclusivity).

**Table 5:** Engagement quality^a^ themes, definitions, and examples.

Theme	Definition	Examples
Relationship-building	A partner perceives relating to others to be a key aspect of engagement.	“We created an environment that was like a support group. It was incredible and I did not expect it.” [Research partner’s interview note] “Nice to meet other people in a similar spot.” [Community partner’s interview note]
Co-learning	A partner perceives the engagement experience as educational (education may come from research partners or from each other).	“Other members mentioned [resources] in examples that I never heard of before.” [Community partner’s evaluation survey] “It is nice to share ideas with people with similar experiences, to understand what has happened to a loved one (or is currently happening).” [Community partner’s evaluation survey]
Satisfaction with prework	A partner expresses the desire for more engagement between design sessions.	“Wished diagrams/materials had been sent out a few days before so he could digest and review.” [Community partner’s interview note] “I like the information that is sent prior to our sessions.” [Community partner’s evaluation survey]
Satisfaction with design activities	A partner evaluates the activities used in sessions, and a partner suggests different activities.	“I did not like the expectation of drawing something, I will never draw for anyone” [Community partner’s interview note] “I would have liked more time to think about what problem I wanted to solve and how I could envision a solution.” [Community partner’s evaluation survey] “I’m excited. I enjoyed the once a month meetings and seeing the development of the app.” [Community partner’s evaluation survey]
Satisfaction with the time allotted	A partner evaluates the time allotted for sessions.	“I wouldn’t mind if they were scheduled for a longer time -- maybe 1.25 or 1.5 hours. It would be nice to not to feel a bit rushed at the end.” [Community partner’s evaluation survey] “I think a couple more sessions would have been beneficial.” [Community partner’s interview note]
Satisfaction with the prototype	A partner evaluates the prototype seen in the session, and a partner makes a suggestion for the prototype.	“Oh my gosh, this [product] would have been so helpful... I’m looking forward to having that go live.” [Community partner’s evaluation survey] “Not having a robust app to see each of the features wasn’t as effective as having a fully functional app.” [Community partner’s evaluation survey]
Influence	This is the partners’ perception of who shaped the final design and how they shaped it.	“Every time we met, the team introduced us to improvements to the modules that were implemented based on what they heard us say.” [Community partner’s reflection survey] “Co-designers directly influenced the design of the product by responding to lead researcher prompts and questions. Those answers were then actualized in design. However, I do feel that the final product would have been more co-designer driven if co-designers had been involved in identifying what type of tool would have been most helpful to them... and identifying what topics should have been discussed at all in design sessions” [Research partner’s reflection survey]
Inclusivity	This is the extent to which partners felt empowered to contribute or actually contributed	“Just like me, every other participant in the design sessions was able to provide ideas, recommendations, and their questions were welcomed.” [Community partner’s reflection survey] “The identities of those involved in the co-design teams played a big role in whose voices were heard... There were a couple of White men who spoke up a lot. Our research team was all White; how did that affect who shared and what they shared?” [Research partner’s reflection survey]

^a^Engagement quality: “The perceptions, assessments and feelings of partners and researchers about the process of engagement” [20].

#### Partner Outcomes

We constructed 4 themes within the domain of partner outcomes: ongoing project interest, gratitude, self-esteem, and sense of meaning. Table 6 presents the themes, definitions, and example quotations relating to partner outcomes.

Community partners expressed interest in the future of the project even after the conclusion of design sessions (eg, asking how they could further contribute; ongoing project interest). Furthermore, partners expressed gratitude for the research partners, for their fellow community partners, or for the opportunity to engage in research (gratitude). Some community partners noted more positive self-appraisal as a result of engagement (self-esteem), while others found meaning in having made a positive contribution to science and to other caregivers (sense of meaning).

**Table 6:** Partner outcomes^a^ themes, definitions, and examples.

Theme	Definition	Examples
Ongoing project interest	Partner expresses interest in continued participation in the project even after the design sessions have ended and partners suggest next steps for the project.	“What happens next? How can we further contribute? Can we see a newer version of the prototype 6 months from now?” [Community partner’s interview note] “I’m looking forward to seeing future progress and if you want any feedback, I’m always willing to participate - even if the funding for gift cards has run out.” [Community partner’s evaluation survey]
Gratitude	Partner describes that engagement in the design sessions inspires gratitude.	“Thank you!! Thank you for including [community partner’s state] in this effort.” [Community partner’s evaluation survey]
Self-esteem	Partner’s self-evaluation becomes more positive as a result of engagement.	“I thought I was valuable, I’m proud of myself, I’ve been a caregiver for years.” [Community partner’s interview note] “Sharing information and realizing we struggled with the same issues. You don’t feel so alone.” [Community partner’s evaluation survey]
Sense of meaning	Partner feels that they have made a meaningful contribution through their engagement.	“The opportunity for participation and to express what we were going through was really powerful.” [Community partner’s interview note] “I learned that I am very fortunate in my situation... Eager to help where needed and to simplify others needs when and where ever possible.” [Community partner’s evaluation survey]

^a^Partner outcomes: “Impact of engagement on the individuals, organizations, and communities partnering in research” [20].

## Discussion

### Principal Findings

This study aimed to (1) understand the context, quality, and outcomes of partners’ engagement experiences in remote PD and (2) learn what aspects of the observed PD approaches facilitated engagement or need to be improved. Much of the evaluation and reflection data were nonspecific but positive, and the effectiveness and satisfaction ratings for each session ranged between 4 and 5 on a 5-point scale, suggesting that many partners had a generally positive experience. Factors associated with engagement context were found to influence engagement, such as the context brought by partners (eg, their identities and their understanding of the project) as well as the context created during the project (eg, the online setting). Within the domain of engagement quality, partners reported greater or lesser degrees of satisfaction with session activities, relationship-building, influence, and inclusivity. Partners who commented on personal outcomes of participation were uniformly positive, noting continued desire to participate in research and a range of psychosocial benefits.

### Preparing Partners for the Remote Context

Although analysis of remote PD is nascent, research on how remote settings affect community-based participatory research (CBPR) is more advanced, and learnings may be applicable to PD [23,27,38-41]. In CBPR, scientific researchers partner with community members, often from high-risk populations, to identify a health concern of interest to community members; build a deep, contextualized understanding of this concern; investigate and cocreate potential interventions to address the concern; and, finally, disseminate and try to sustain these interventions within the community [42]. Previous CBPR notes that the remote approach comes with technological challenges, including differing levels of familiarity with teleconferencing software and the development of “Zoom fatigue” [27]. In this study, research and community partners did express some wariness about using teleconferencing software for PD. Research partners noted challenges with translating traditionally in-person design processes to the remote space, noting fewer opportunities for potentially generative side conversations between partners. However, the remote setting did not appear to prohibit colearning or relationship development, although experimental study designs are needed to confirm this finding.

Existing literature in the fields of CBPR and patient engagement highlights the necessity of adequately preparing community partners to engage in research [28,43]. In remote PD, it is not commonplace to provide partners with this thorough background information. In this study, for projects 1 to 4, community partners were provided with a study information sheet, a consent and screening call, and an introductory session in which the description of the project lasted for up to 20 minutes. However, our findings related to project and role understanding suggest that, for some partners, this preparation was insufficient. For some partners, poor understanding may have led to reduced participation. Future remote PD studies should integrate community partners from the project inception, including defining the problem of greatest interest to community partners, coauthoring the approach, and brainstorming how to best use the remote space. Furthermore, during and between design sessions, researchers may use an approachable, predefined process to clarify partners’ understanding of the project’s goals and partner roles. To this end, the remote context allows researchers to easily distribute links to surveys, conduct polls, and receive public and private chats. Furthermore, researchers should prepare materials in multiple formats (eg, written, auditory, and image-based materials) and provide alternative ways of phrasing concepts to maximize community partner comprehension [44].

### Activities to Promote Remote Engagement

Community and research partners were mixed in their evaluation of inclusivity and influence. In contrast to previous research, community partners in this study reported seeing their contributions reflected in the design and rated themselves as having had a strong influence on the design [45]. However, echoing previous research, some research partners in this study felt that the design process was not sufficiently led by the community partners, perceiving community partners as providing feedback on the design but not leading design efforts [46]. For PD products to be the best possible fit for their intended populations and for PD to hold positive meaning for community and research partners alike, inclusivity and equality of influence must be prioritized in PD as they are in CBPR [43,47]. It is recommended that research and community partners alike speak candidly about the potential for power differentials present in the broader culture (eg, among those of different genders, races, disabilities, levels of education, and project roles) to manifest in remote spaces as they do in physical spaces [43,48-51]. Partners should speak frequently about how to create and maintain group structures and norms that combat power imbalances. Conversations such as these require trust. Thus, the concepts of inclusivity and influence should be introduced to partners before the design process begins, senior research partners should model these conversations early, and community partners should be encouraged to contribute as they feel it is safe to do so. For community and research partners who do not feel safe voicing these concerns aloud, the remote context allows for the distribution of anonymous surveys.

Community partners ranged in their satisfaction with the design sessions, including with the presession work, the design activities that occurred in the session, the time allotted, and the status of the prototype in the final session. While the activities used in these remote PD study are consistent with those of in-person studies, it is recommended that future studies use more nuanced evaluation methods to determine which remote activities were perceived as most effective and enjoyable by partners [18]. When designing the structure of the study, research partners sought to make modest requests of community partners to avoid fatigue or study attrition, which are problems that have been documented in other projects conducted remotely [27]. However, qualitative analyses revealed that community partners enjoyed the presession work, often requesting that more materials and reflection questions be sent out in advance. Community partners often commented that 1-hour sessions felt too short for the task at hand. This suggests that community partners are prepared for more intensive engagement in remote design. In future remote studies, online process checks may be used to gather real-time feedback about the workload and time commitment, and this feedback should be reflected in changes to the study timeline.

### Maximizing Community Partner Outcomes

Finally, our results concur with CBPR literature, suggesting that remote PD has the potential to create positive outcomes for partners, including empowerment for partners and positive impacts on their health [47]. Community partners in this study expressed gratitude for their involvement, greater self-esteem, and a sense of purpose. Furthermore, community partners expressed interest in continued remote involvement with the design and implementation of the tool. When designing this study, research partners underestimated the extent to which involvement could provide a meaningful experience for community partners. Going forward, it is recommended to conceptualize remote PD as a process that may help community partners (especially those who are isolated due to geographic or physical limitations) achieve developmental tasks, such as intimacy (closeness with others), generativity (benefiting future generations), and integrity (having lived a meaningful life) [52]. Involving community partners to this extent requires compensation for their participation. This goes beyond financial compensation to include remote training relevant to the research topic and methods, facilitating remote networking between partners, distance collaboration on manuscripts, and copresentation at conferences [28,53-55]. Furthermore, for designs that reach the commercial marketplace, community partners should share any resulting profits.

### Recommendations for Remote PD Research

Overall, these results point to high satisfaction with our remote PD processes, as well as specific ways in which processes can be changed to improve partner engagement and avenues for maximizing positive partner outcomes. Recommendations for engaging community and research partners in remote PD are listed in Table 7.

**Table 7:** Integrated results and strategies for engaging research and community partners in remote participatory design (PD) research.

Qualitative theme or quantitative finding	Strategies for engagement in remote PD
Engagement context: identity influence; project understanding; role understanding	From project inception, include partners in defining the problem to be solved and the approach to be used (to facilitate project understanding) and in defining their role (to facilitate role understanding). Include partners of different gender identities, sexual orientations, races, disabilities, levels of education, and levels of academic seniority. During the PD process, use live discussion, surveys, polls, and the chat feature to perform remote “process checks” to ascertain partners’ project and role understanding.
Engagement context: technological context	From project inception, involve partners in defining how to use the remote space, including specifying the technologies (eg, teleconferencing and online whiteboards) and technological functions (eg, chat and poll) with which they are comfortable. Outside of PD sessions, assist interested partners in gaining technological proficiency through 1:1 or small group meetings.
Engagement quality: satisfaction with prework; satisfaction with design activities; satisfaction with the time allotted; high quantitative satisfaction ratings with design sessions	From project inception, involve partners in defining the amount of prework desired or needed, imagining design activities, and designating the time allotted for activities. During the PD process, use live discussion, surveys, polls, and the chat feature to solicit feedback on changes that should be made to the design process. After the PD process, ask sensitive and specific evaluation and reflection questions to understand their satisfaction with different aspects of the design process.
Engagement quality: satisfaction with the prototype; influence; inclusivity; quantitative difference between community and research partners’ estimations of who influenced the final product	Encourage dual roles, that is, equipping community partners to facilitate the research process or hiring research partners who are members of the community of interest. From project inception, emphasize and define processes for sharing power, especially in remote spaces (eg, raising hands and inviting quieter partners to share). During the PD process, define processes for intervening when power is not being shared (ie, what to say, how to say it, whom to say it to, and through what medium). During the PD process, make available a link to an anonymous survey in which partners can submit feedback and ideas on inclusion and power-sharing.
Engagement quality: relationship-building; co-learning	From project inception, emphasize and expand the potential for building connections between partners (eg, with consent, facilitating the exchange of contact information). Elicit caregivers’ areas of expertise or experience within caregiving and facilitate knowledge exchange within and outside of sessions.
Partner outcomes: ongoing project interest; gratitude; self-esteem; sense of meaning	At project conclusion, invite community partners to participate in future aims of the project or related projects. Budget for involving community partners in the dissemination of findings, including conference travel, coauthorship of manuscripts, and bringing findings to communities.

### Limitations and Future Research

This analysis had some limitations. First, across design sessions, research and community partners were disproportionately White and cisgender individuals. This lack of diversity may have led to an incomplete understanding of how concepts such as trust, inclusivity, and influence manifest in remote PD [56]. Identity-based barriers to engagement were seldom mentioned by partners in this study. However, future research should investigate how to meaningfully engage partners with physical disabilities, as certain partners with disabilities may uniquely benefit from the remote setting (remote PD requires less mobility) while others may find it more complicated (remote PD is often dependent on visual media) [57]. Second, evaluation and reflection data are only as accurate as partners felt safe to provide. It is vital to establish and reinforce trust between research and community partners and to provide anonymous remote formats for providing feedback so that partners do not feel pressured to provide socially desirable feedback. Third, although community partner evaluation data were available for all studies, research partners only completed evaluations in 1 study (project 4), and reflection data were only gathered from research and community partners in 1 study (project 1). It is not yet commonplace to gather evaluation and reflection data from research and community partners in PD studies [18]. However, to facilitate growth in the science of improving remote PD engagement, future studies should more rigorously gather evaluation and reflection data from all partners across domains, such as engagement context, engagement quality, and partner outcomes. Fourth, the design of this study did not allow researchers to directly compare in-person PD to remote PD on measures of engagement; future research should adopt experimental designs to this end. Finally, the evaluation and reflection data reflect only short-term partner outcomes of engagement. Future work should evaluate whether heightened senses of meaning, gratitude, and community persist long term as a result of partnering in remote PD.

### Conclusions

This analysis of 4 remote PD studies points to ways in which remote PD processes must be more thoroughly evaluated and, where indicated, changed to enhance context, quality, and outcomes for partners. While the remote context was sometimes a barrier to collaboration, it did not seem to prohibit colearning or the development of relationships. Indeed, many of our findings echo previous work on in-person PD, indicating few losses specific to remote PD. It may be that the gains associated with remote PD, including the reduced expense, reduced travel time, and greater potential for engaging underserved populations, surpass the losses of the remote setting. Future work should not just evaluate the adaptation of in-person activities to remote settings but rather more thoroughly reimagine PD as a remote process with the unique affordances of increasingly sophisticated remote environments. Finally, our results suggest that community partners are open to a more time-intensive commitment to remote PD. Future research should systematically examine whether more intensive involvement of community and research partners in remote PD yields better outcomes for research, partners, and the communities that the research aims to serve.

## Data Availability

The datasets generated during and analyzed during this study are available from the corresponding author on reasonable request.

## Abbreviations

ADRD: Alzheimer disease and related dementias
CBPR: community-based participatory research
PCORI: Patient-Centered Outcomes Research Institute
PD: participatory design
