# Correction: Investigating the Best Practices for Engagement in Remote Participatory Design: Mixed Methods Analysis of 4 Remote Studies With Family Caregivers

**DOI:** 10.2196/69714

**Published:** 2024-12-31

**Authors:** Anna Jolliff, Richard J Holden, Rupa Valdez, Ryan J Coller, Himalaya Patel, Matthew Zuraw, Anna Linden, Aaron Ganci, Christian Elliott, Nicole E Werner

**Affiliations:** 1 Department of Health & Wellness Design School of Public Health - Bloomington Indiana University Bloomington Bloomington, IN United States; 2 Department of Systems and Information Engineering University of Virginia Charlottesville, VA United States; 3 Department of Public Health Sciences University of Virginia Charlottesville, VA United States; 4 Department of Pediatrics School of Medicine and Public Health University of Wisconsin - Madison Madison, WI United States; 5 Health Systems Research Center for Health Information and Communication Richard L Roudebush Veterans Affairs Medical Center United States Department of Veterans Affairs Indianapolis, IN United States; 6 CareVirtue Technologies San Diego, CA United States; 7 Department of Industrial & Systems Engineering University of Wisconsin - Madison Madison, WI United States; 8 Department of Visual Communication Design Herron School of Art & Design Indiana University Indianapolis Indianapolis, IN United States

In “Investigating the Best Practices for Engagement in Remote Participatory Design: Mixed Methods Analysis of 4 Remote Studies With Family Caregivers,” (J Med Internet Res 2024;26:e60353), the authors noted one error.

The following author’s affiliation was listed as:

Himalaya Patel^5^

^5^Health Systems Research Center for Health Information and Communication, Richard L Roudebush Veterans Affairs Medical Center, United States Department of Veterans Affairs, Indianapolis, IN, United States

The author’s affiliation has been corrected to:

Himalaya Patel^1,5^

^1^Department of Health & Wellness Design, School of Public Health - Bloomington, Indiana University Bloomington, Bloomington, IN, United States

^5^Health Systems Research Center for Health Information and Communication, Richard L Roudebush Veterans Affairs Medical Center, United States Department of Veterans Affairs, Indianapolis, IN, United States

Additionally, middle initial J has been added to author name Ryan J Coller. The name has been changed from:

Ryan Coller

to

Ryan J Coller

The correction will appear in the online version of the paper on the JMIR Publications website on December 31, 2024, together with the publication of this correction notice. Because this was made after submission to PubMed, PubMed Central, and other full-text repositories, the corrected article has also been resubmitted to those repositories.

